# Scalp necrotic wound and hyperinflammatory shock related to COVID‐19: Topical sucralfate as a promising topical agent

**DOI:** 10.1111/iwj.13936

**Published:** 2022-08-24

**Authors:** Zahra Pourmoghaddas, Fereshte Rastegarnasab, Ali Mohammad Sabzghabaee, Bahareh Abtahi‐Naeini

**Affiliations:** ^1^ Pediatric Cardiovascular Research Center, Cardiovascular Research Institute Isfahan University of Medical Sciences Isfahan Iran; ^2^ Pediatrics Infectious Diseases Department Isfahan University of Medical Sciences Isfahan Iran; ^3^ Student Research Committee Isfahan University of Medical Sciences Isfahan Iran; ^4^ Isfahan Clinical Toxicology Research Center Isfahan University of Medical Sciences Isfahan Iran; ^5^ Pediatric Dermatology Division of Department of Pediatrics, Imam Hossein Children's Hospital Isfahan University of Medical Sciences Isfahan; ^6^ Skin Diseases and Leishmaniasis Research Center Isfahan University of Medical Sciences Isfahan Iran

Dear Editors,

A 1‐year‐old previously healthy boy presented with a 5‐day fever, diarrhoea, vomiting, and clinical signs and symptoms of shock associated with positive serologic tests for COVID‐19 and increased inflammatory markers. He was admitted with a diagnosis of multisystem inflammatory syndrome in children (MIS‐C) in association with COVID‐19. Symptoms of shock were treated with fluid therapy, epinephrine, and milrinone during hospitalisation. The patient underwent pulse methylprednisolone, 30 mg/kg for two doses, and intravenous immunoglobulin (IVIG) therapy 2 g/kg. During the 3 days of hospitalisation, the patient developed an abrupt area of epinephrine‐induced vasoconstriction followed by persistent ischaemic dusky‐red discoloration on the scalp for 10 hours due to the unavailability of an ischaemic reversal event using phentolamine and nitroglycerin paste. The patient was consulted by the paediatric surgeon and paediatric dermatologist who considered the condition as epinephrine‐induced dermal necrosis. Therapy including local wound care and topical sucralfate cream was used at the site of the necrotic lesion. No cutaneous sequel was observed on day 20 of hospitalisation (Figure 1).

**FIGURE 1:**
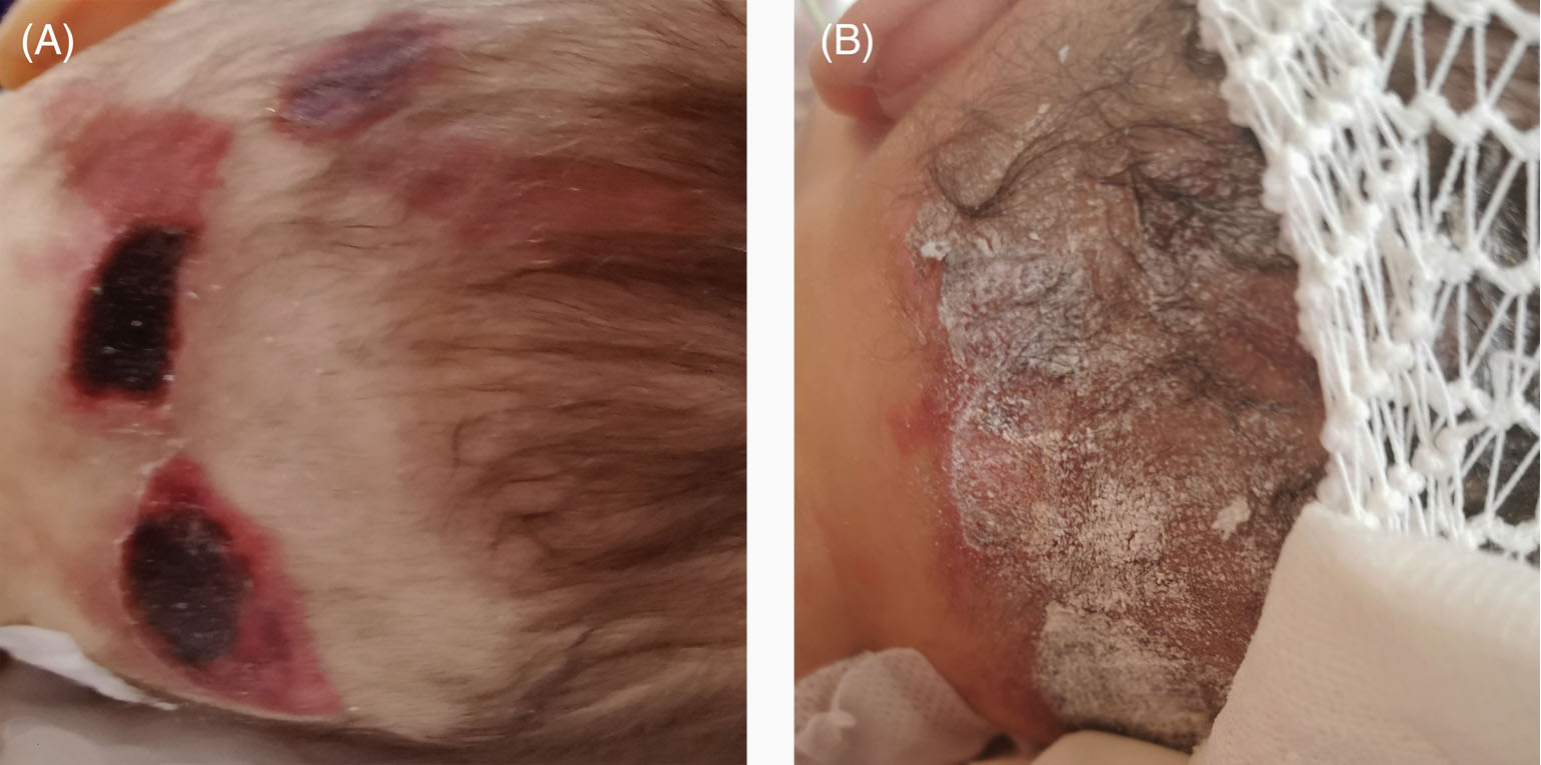
Epinephrine‐induced dermal necrosis. Before the treatment (A), after the treatment with topical sucralfate (B)

For topical preparation of topical 8% sucralfate, 16 tablets of sucralfate (each 500 mg) were crushed and pulverised to a fine powder. Next, it was wetted with 10 mL of glycerin and levigated with a blended formulation of Zinc oxide (20%) and petrolatum (Iroxcare Inc.) to a total of 100 mL.

The current case highlights the specific promising clinical benefits of topical sucralfate for dermal necrosis treatment. In our experience, topical sucralfate can be a promising safe agent for treating epinephrine‐induced tissue necrosis.

Also, our case emphasises the importance of early signs and symptoms of vasopressin‐induced extravasation to recognize extravasation promptly. In addition, this case highlights the safe use of vasopressin and specific antidotes for extravasation to prevent more tissue loss.

Rarely, extravasation injuries can present with skin breakdown. Consequently, they may lead to serious complications such as skin necrosis, gangrene, secondary infection, permanent nerve damage, contracture of affected limbs, and amputation if left untreated.^1^, ^2^ Different predisposing factors can cause this condition. The major causes of this issue are mechanical factors (eg, poor condition of veins, unsuitable size of catheter, unstable catheter, and patient activity), physiological factors (eg, clot formation above the cannulation site or at the catheter tip), and pharmacological factors (eg, pH, osmolarity, vasoactivity of the medication, and cytotoxicity).^3^, ^4^, ^5^ Children mostly have multiple risk factors, increasing susceptibility to extravasation.^6^ Extravasation injuries associated with vasoconstrictive agents can lead to ischaemic necrosis.^5^ Critical conditions such as shock and underlying conditions of endothelial damage are potential predisposing factors for the development of extravasation injuries. Management of extravasation injuries is controversial.^7^ Phentolamine is an FDA‐approved antidote and the first choice for treating the extravasation induced by vasoconstrictive drugs. It competitively works as an α‐receptor antagonist and reverses ischaemic changes by vasodilation. It is most effective when injected subcutaneously within 12 hours of extravasation.^8^ Topical nitroglycerin has been reported successful in the treatment of vasoactive‐induced extravasation.^9^ In addition, supportive care like warm compress and wound care can help better healing. Eventually, surgical debridement may be needed in case of skin necrosis.^10^

In our patient, the urgent and emergent use of phentolamine and topical nitroglycerin was not available. Also, surgical debridement was not performed due to the general condition and bleeding diathesis secondary to MISC. Topical sucralfate, an aluminium salt of sucrose octasulfate, has been successfully used in different mucocutaneous conditions. For instance, it is used in inflammatory conditions (eg, post‐radiotherapy reaction, peristomal wound reaction, oral lesions, ocular lesions, rectal lesions, and dermatitis), burns, and cutaneous ulceration. Suggested mechanisms of action for topical sucralfate include decreasing cell apoptosis and improving growth factor bioavailability. It helps tissue growth, regeneration, and repair by improving blood flow, cell proliferation, and repair via the connection of growth factors to tissues. Sucralfate is considered as a safe drug in terms of adverse effects.^11^, ^12^, ^13^

## CONFLICT OF INTEREST

The authors declare no potential conflict of interest.

## Data Availability

The data that support the findings of this study are available on request from the corresponding author.

## References

[1] Reynolds PM , MacLaren R , Mueller SW , Fish DN , Kiser TH . Management of extravasation injuries: a focused evaluation of noncytotoxic medications. Pharmacother J Hum Pharmacol Drug Ther. 2014;34(6):617‐632. 10.1002/phar.1396 24420913

[2] Ghanem AM , Mansour A , Exton R , et al. Childhood extravasation injuries: improved outcome following the introduction of hospital‐wide guidelines. J Plast Reconstr Aesthet Surg. 2015;68(4):505‐518. 25618570 10.1016/j.bjps.2014.12.029

[3] Hadaway L . Infiltration and extravasation. Am J Nurs. 2007;107(8):64‐72. 10.1097/01.NAJ.0000282299.03441.c7 17667395

[4] Goolsby TV , Lombardo FA , Extravasation of chemotherapeutic agents: prevention and treatment. Seminars in oncology. Elsevier; 2006. 10.1053/j.seminoncol.2005.11.007 16473651

[5] Doellman D , Hadaway L , Bowe‐Geddes LA , et al. Infiltration and extravasation: update on prevention and management. J Infus Nurs. 2009;32(4):203‐211. 19605999 10.1097/NAN.0b013e3181aac042

[6] Paquette V , McGloin R , Northway T , DeZorzi P , Singh A , Carr R . Describing intravenous extravasation in children (DIVE Study). Can J Hosp Pharm. 2011;64(5):340‐345. 22479086 10.4212/cjhp.v64i5.1069 PMC3203826

[7] Goutos I , Cogswell LK , Giele H . Extravasation injuries: a review. J Hand Surg. 2014;39(8):808‐818. 10.1177/1753193413511921 24401738

[8] Le A , Patel S . Extravasation of noncytotoxic drugs: a review of the literature. Ann Pharmacother. 2014;48(7):870‐886. 24714850 10.1177/1060028014527820

[9] Denkler KA , Cohen BE . Reversal of dopamine extravasation injury with topical nitroglycerin ointment. Plast Reconstr Surg. 1989;84(5):811‐813. 2510208 10.1097/00006534-198911000-00017

[10] Alexander CM , Ramseyer M , Beatty JS . Missed extravasation injury from peripheral infusion of norepinephrine resulting in forearm compartment syndrome and amputation. Am Surg. 2016;82(7):E162‐e163. 27457846

[11] Masuelli L , Tumino G , Turriziani M , Modesti A , Bei R . Topical use of sucralfate in epithelial wound healing: clinical evidences and molecular mechanisms of action. Recent Pat Inflamm Allergy Drug Discov. 2010;4(1):25‐36. 19832693 10.2174/187221310789895649

[12] Abtahi‐Naeini B , Saffaei A , Sabzghabaee AM , et al. Topical sucralfate for treatment of mucocutaneous conditions: a systematic review on clinical evidences. Dermatol Ther. 2022;35(4):e15334. 35080090 10.1111/dth.15334

[13] Saneian H , Mehrannia A , Sabzghabaee AM , Feizi A , Famouri F , Abtahi‐Naeini B . Topical Sucralfate for prevention of peristomal wound reaction related to percutaneous endoscopic gastrostomy in children: a randomized controlled trial. Dermatol Ther. 2022;e15729. 10.1111/dth.15729 35871473

